# An Experimental Validated Computational Method for pKa Determination of Substituted 1,2-Dihydroxybenzenes

**DOI:** 10.3389/fchem.2018.00208

**Published:** 2018-07-13

**Authors:** Romina Romero, Pablo R. Salgado, César Soto, David Contreras, Victoria Melin

**Affiliations:** ^1^Technological Development Unit, University of Concepcion, Coronel, Chile; ^2^Engineering and Environmental Biotechnology Group, Faculty of Environmental Sciences and Eula-Chile Center, University of Concepcion, Concepción, Chile; ^3^Department of Analytical and Inorganic Chemistry, Faculty of Chemical Sciences, University of Concepcion, Concepción, Chile; ^4^Biotechnology Center, University of Concepcion, Concepción, Chile

**Keywords:** dihydroxybenzene, pKa, catechols, catecholamines, Hammett constant and acidity constant

## Abstract

1,2-dihydroxybenzenes (DHBs) are organic compounds which are widely studied as they are applied to advanced oxidation processes (AOPs). These compounds are also related to the development of oxidative stress, wood biodegradation, and neuronal disease in humans. DHBs are metal ligands with pro-oxidant and antioxidant properties. These activities are related to their chelation properties and a consequence of the deprotonation of their hydroxyl groups. In literature, there are several pKa values for the hydroxyl groups of DHBs. These values vary depending on the experimental conditions or the algorithm used for calculation. In this work, an experimentally validated computational method was implemented in aqueous solution for pKa determination of 24 DHBs. The deprotonation order of the hydroxyl groups in DHB was determined observing a selective deprotonation, which depended on the ability of the substituent to donate or withdraw electron density over the ring.

## Introduction

**Figure 1:**
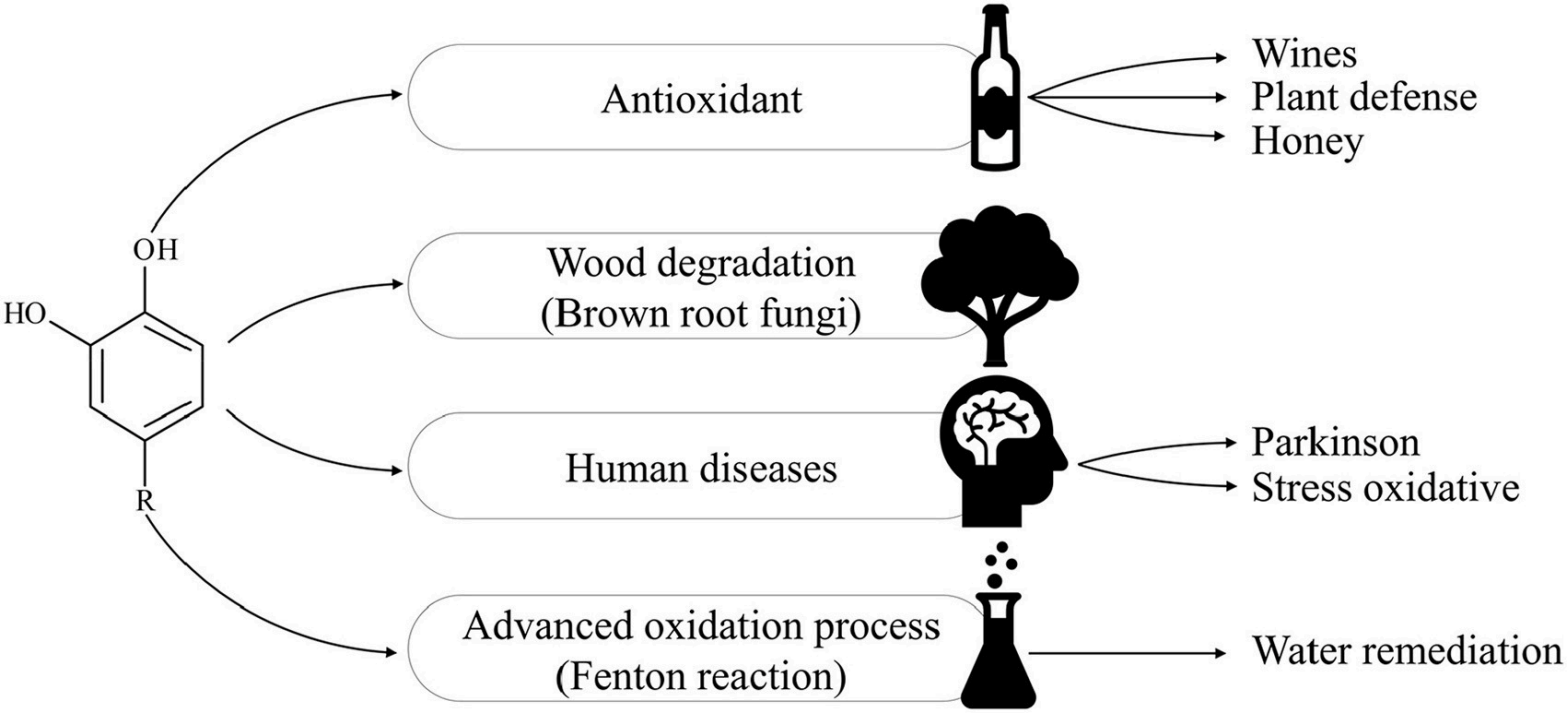
Applications and biochemical processes in which 1,2-DHB have been studied.

1,2-dihydroxybenzenes (DHBs) are organic compounds that exhibit antioxidant and/or pro-oxidant activities in several biological systems (Iwahashi et al., 1989; Moran et al., 1997; Nakamura et al., 2000; Schweigert et al., 2001; Gomes et al., 2003; Zang et al., 2003; Ohara et al., 2006; Prousek, 2007) depending on the reaction conditions (Salgado et al., 2013; Melin et al., 2015). These compounds have actively participate in important biochemical processes, which are shown in Figure 1.

The antioxidant properties of DHBs are based on its radical scavenging properties (disrupting the radical chain reaction propagation) or iron chelating (disrupting the initiation step of the radical chain reaction) (Perron and Brumaghim, 2009). This role is important in plants' defense mechanisms against pathogens (Lukasik, 2007) and these antioxidant properties are present in different kinds of food, such as wine, honey, tea and blueberries (Ricci et al., 2018; Santhakumar et al., 2018; Yang et al., 2018).

Pro-oxidant activities of DHBs have also been widely studied in wood biodegradation caused by brown and white rot fungi. DHBs are produced by fungi as a type of non-enzymatic degradation mechanism that could penetrate through the wood cell wall matrix and degrade the wood by redox reactions (Goodell et al., 2006). In humans, dopamine, a kind of DHB, has been related to the progression of Parkinson's disease due to oxidative stress (Kuhn et al., 1999). In addition, the oxidation of catecholamines by reperfusion could generate oxidative stress and increase the cellular damage at cardiac level (Bindoli et al., 1992; Schweigert et al., 2001). Pro-oxidant properties of these compounds are based on their ability to reduce Fe(III) and O_2_ to Fe(II) and HO_2_, respectively. In the same way, DHBs have been used to increase the oxidant properties of Fenton's reagent on recalcitrant compound; thus, these compounds also have potential applications in advanced oxidation processes (AOPs) (Rodríguez et al., 1999; Rodriguez et al., 2003; Rodríguez et al., 2004).

The pro-oxidant and antioxidant activities of the DHBs are closely related to the formation of complexes with metals, which require the deprotonation of the hydroxyl groups (Avdeef et al., 1978; Hynes and Ó Coinceanainn, 2001; Hynes and O'Coinceanainn, 2004). Moreover, the kind of complex formed between DHB and metals is related to the pKa values of DHB (Jameson and Wilson, 1972; Pizer and Babcock, 1977; McBride et al., 1988). Considering this, and the wide application of DHBs, knowledge of the pKa values of each hydroxyl group becomes necessary.

For some commercially available DHB, pKa values have been reported under different methods or experimental conditions. This leads to situations where the reported pKa values show significant differences. Table 1 shows that in the case of catechol, there are several reports of pKa. These works assign values between 9.14 (Nurchi et al., 2009) and 9.43 (Park, 1963) pH units for the first pKa, and values between 11.49 (Pizer and Babcock, 1977) and 13.8 pH units (Nurchi et al., 2009) for the second pKa. Compounds such as 3,4-dihydroxybenzoic acid, 4-nitrocatechol, caffeic acid, and dopamine are also under similar situations.

**Table 1:** pKa values for the hydroxyl group reported for different DHBs.

**Compound**	**pKa_1_**	**pKa_2_**	**References**
1,2-dihydroxybenzene (catechol)	9.14	13.80	Nurchi et al., 2009
	9.15	11.23	Timberlake, 1957
	9.21	11.70	Antikainen and Witikainen, 1973
	9.22	13.00	Avdeef et al., 1978
	9.24	13.00	Charkoudian et al., 2006
	9.27	11.49	Pizer and Babcock, 1977
	9.33	12.62	Park, 1963
	9.34	13.24	Evanko and Dzombak, 1998
	9.37	13.70	Slabbert, 1977
	9.43	13.00	Park, 1963
3,4- dihydroxybenzoic acid	8.64	13.13	Beltran et al., 2003
	8.67	11.74	Jovanovic et al., 1994
	8.82	13.20	Slabbert, 1977
1,2-Dihydroxy-4-nitrobenzene (nitrocatechol)	6.62	10.75	Aydin et al., 1997
	6.65	10.80	Avdeef et al., 1978
	6.69	10.57	Pizer and Babcock, 1977
	6.7	10.31	Nurchi et al., 2009
	6.84	11.1	Slabbert, 1977
3-(3,4-Dihydroxyphenyl)-2-propenoic acid (caffeic acid)	8.14	13.16	Erdemgil et al., 2007
	8.47		Fazary and Ju, 2008
	8.672	12.6	Adams et al., 2002
	8.83		Ozkorucuklu et al., 2009
4-(2-aminoethyl)benzene-1,2-diol (dopamine)	8.86	10.31	Antikainen and Witikainen, 1973
	8.89	10.41	Kiss et al., 1989
	8.96	10.5	Rajan et al., 1972
	9.59	13.11	Charkoudian et al., 2006

There are different methods used to calculate the pKa value for a compound. In general, these can be divided into experimental or computational methods. The experimental methods involve the monitoring of a physical property that varies with protonation of the molecule (Reijenga et al., 2013). Computational methods generally determine the pKa values by using thermodynamic cycles (Pliego, 2003). Computational methods have the advantage of having relatively good accuracy, low cost (Pathare et al., 2014), and facilitate determinations of pKa values at pH ranges where the compound lose solubility. However, to obtain good results, it is highly important to perform calculations using an adequate model of solvation (Sharma and Kaminski, 2012) and possess experimental data to validate the computational results.

It is interesting to obtain the pKa values of DHBs by reproducible and validated methods in aqueous solution, since these compounds have a wide range of applications in biological systems. In the literature, it is possible to find calculated pKa values for these molecules in dimethyl sulphoxide (DMSO) (Zhu et al., 2010), however, it is not possible to infer the same behavior in aqueous solution. Hence, in this work, a DFT calculation was implemented to predict pKa values of a group of 24 DHBs, which were validated through experimental determination using spectrophotometric methods.

Furthermore, in this work, the pKa values of DHBs were systematically determined in order to obtain reproducible values in aqueous solution. The pKa values for each hydroxyl group in the DHB were determined by computational methods and associated with experimental values.

## Methodology

### Reagents

**Figure 2:**
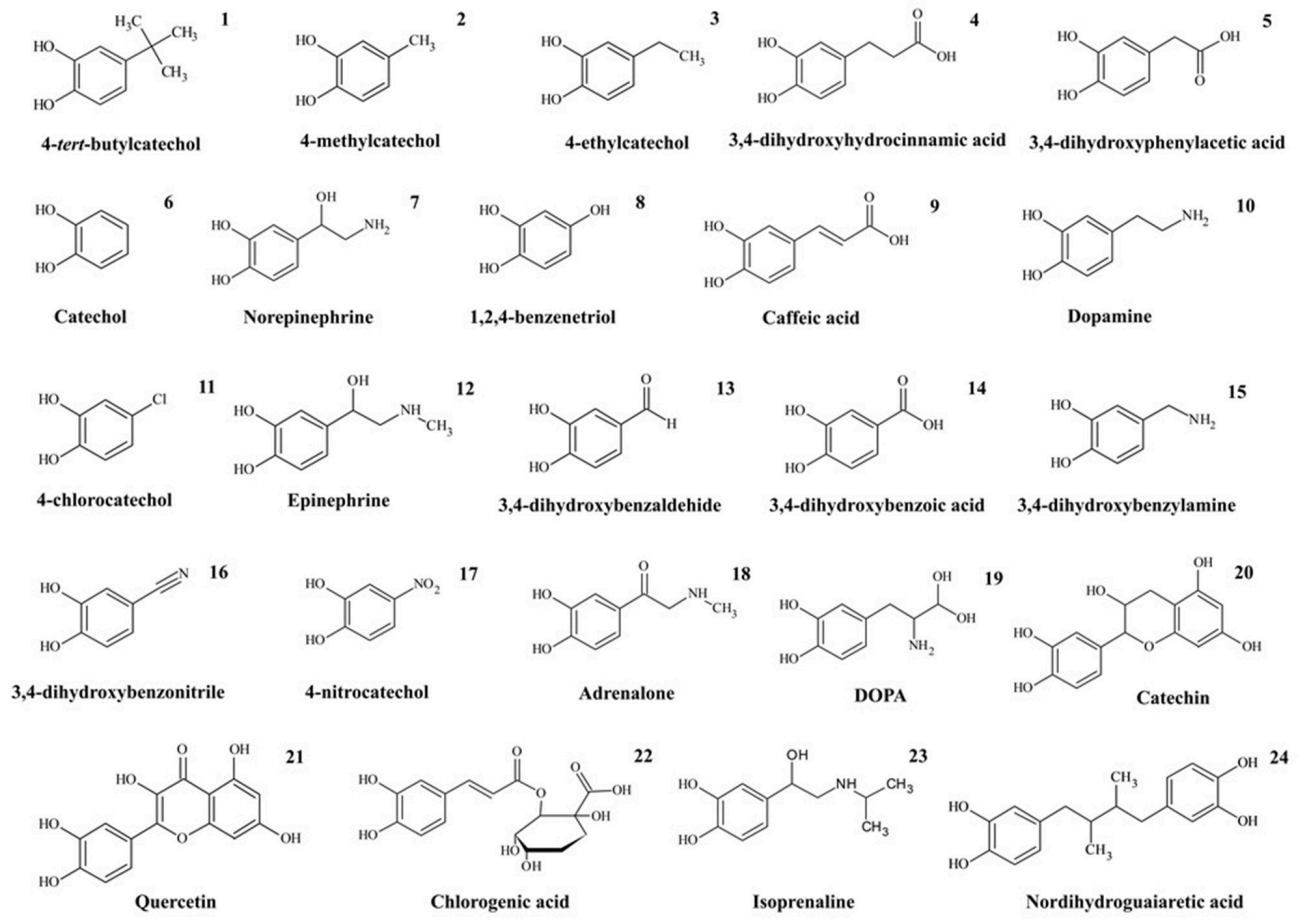
1,2-dihydroxybenzenes studied.

All reagents were purchased from Sigma-Aldrich (Sigma-Aldrich, Germany) at their highest available purity and were used without any further purification steps. The DHBs studied are detailed in Figure 2. The H_3_BO_3_, H_3_PO_4_, and CH_3_COOH used for the Britton-Robinson buffer preparation were supplied by Merck (Merck, Germany).

### Spectrophotometric pKa determination

The DHBs were prepared in Britton-Robinson buffer at a pH value between 2.0 and 14.0, because this pH range is useful for biological systems and AOPs studies. The pH of each solution was adjusted just before each reaction with NaOH using a 3 Start Thermo Orion pH meter (Thermo Fisher Scientific, USA). The final concentration in the cuvette was 1.0 mmol L^−1^ for each DHB.

The absorption spectrum of the solution with the DHB at different pH was determined using a UV-Vis diode array spectrophotometer, Agilent 8453 (Agilent Technologies, USA). All the experimental data were performed by triplicate under argon atmosphere at room temperature (approximately 25°C).

The spectrum obtained at different pH were plotted and analyzed to determine the pKa from the isosbestic points where the Henderson-Hasselbach equation (Equation 1) can be applied (Fernandes Previdello et al., 2006). When an isosbestic point was detected in the plot, the wavelengths with maximum absorbance near but at different sides of this point were selected. Then the absorbance obtained at the selected wavelengths and at different pH was plotted. The point where both lines intersect determines the value of pKa.

(1) $$\begin{array}{l} \text{pH}=\text{p}{\text{K}}_{\text{a}}+\text{log}\left(\left[{\text{HL}}^{-}\right]/\left[{\text{H}}_{2}\text{L}\right]\right) \end{array}$$

Derivative spectrophotometry (D.S) was used when the isosbestic point was not found in the data obtained directly from the spectrophotometer. For finding the derivative of each spectra, the software ChemStation (Agilent Technologies, USA) was used. The first and second spectrum derivative were previously analyzed before applying any of the methods described below to determine the pKa value.

If the isosbestic point was not found in the original or derivative data, zero-crossing line method was used (Seleim et al., 2009). For this, two wavelengths were searched with the derivatives of the spectra. The first wavelength corresponds to a zero signal at pH “X” while at another, pH “Y” was obtained as a high absorbance signal. Afterwards, in another wavelength, the value of absorbance for pH “Y” was zero while the high absorbance was obtained for pH “X.” Then, the absorbance of the solution at different pH at each selected wavelength was plotted and the pKa value was determined from the point where both lines intersect.

If the pKa value cannot be calculated using a zero-crossing line, a baseline to peak method was applied, which searchedfor a wavelength related to a maximum where the absorbance signal was increasing as the pH increased (Erskine and Bobbitt, 1989). In that case, the relative amount of the basic form of a DHB at that wavelength (M) was obtained by subtracting the signal of the solution with DHB at a specific pH from the blank signal, whereas the relative amount of the acid form of the same DHB (N) was determined by subtracting the signal obtained for M with the signal of the most basic solution with DHB. From the Y axis intercept of the plot, log(M/N) v/s pH was obtained pKa (Equation 2) (Erskine and Bobbitt, 1989).

(2) $$\text{p}{\text{H}}_{\left[\text{X}\right]}\;=\text{p}{\text{K}}_{\text{a }}\;+\text{ log }\left(\frac{\text{M}}{\text{N}}\right)$$

(3) $$\text{p}K_{a\;}\;=\text{p}{\text{H}}_{1}\;-\;\log\frac{\left(10^{b}\;-10^{a}\right)\;A_{1}\;+\;\left(1\;-10^{b}\right)\;A_{2}+(10^{a}\;-1)\;A_{3}}{\left(10^{a}\;-10^{b}\right)\;A_{1}\;+\left(10^{a+b}\;-10^{a}\right)\;A_{2}\;+\left(10^{b}\;-10^{a+b}\right)\;A_{3}}$$

The Seok method (Seok et al., 1995) was used if the pKa could not be determined by any previously detailed method. For this, search a wavelength related to a maximum where the absorbance signal was increasing as the pH increased. At that wavelength, three different pH and their respective absorbance (A_i_) were selected. The pKa was calculated using Equation (3).

Where pH_1_ was the lowest pH and pH_3_ the highest pH selected, “a” was the difference between pH_2_ and pH_1_, “b” the difference between pH_3_ and pH_1_, A_1_ was the absorbance at pH_1_ at the selected wavelength, A_2_ was the absorbance at pH_2_ and A_3_ was the absorbance for pH_3_.

### Computational pKa determination

**Figure 3:**
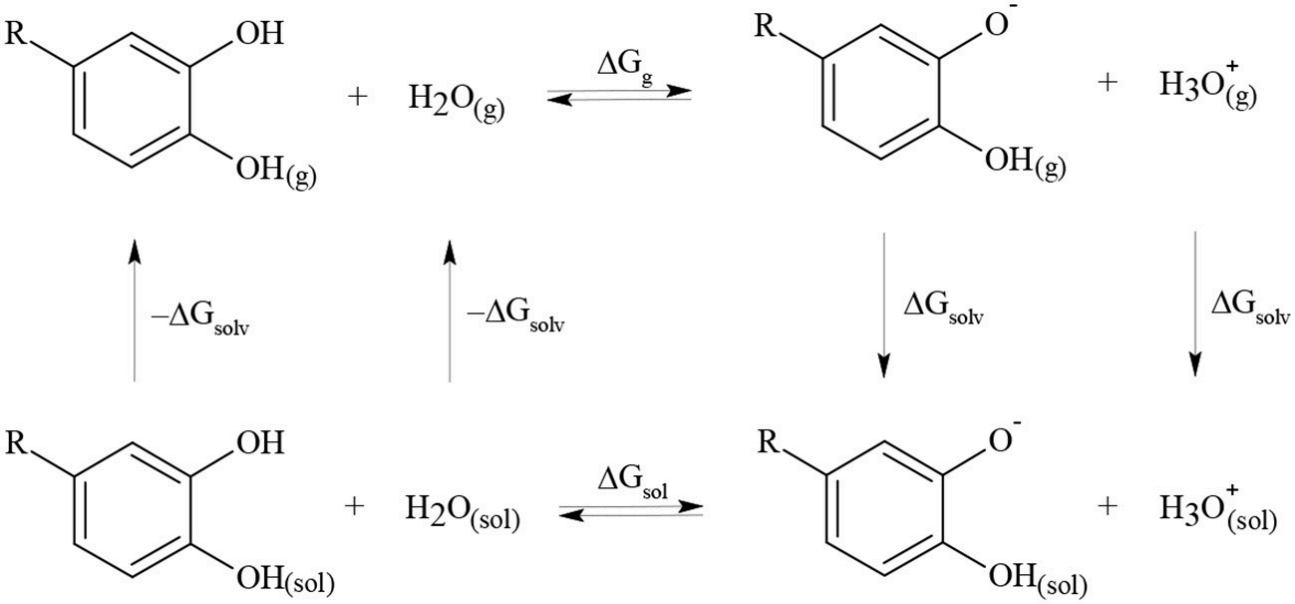
Thermodynamic cycle used to determine pKa value for different DHBs.

The pKa values were determined based on the thermodynamic cycle proposed by Pliego (2003) as shown in Figure 3.

The electronic structures of all species in the thermodynamic cycle were optimized using Gaussian 09 (Frisch and Trucks, 2009). According to previous reports for catechol, the density functional theory (DFT) with B3LYP function and 6-311+G(d,p) basis set was used (Altarawneh et al., 2010). The absence of imaginary frequencies in the optimized structure verified that the structure represented a local minimum. All calculations were performed at 298.15 K and 1.000 atm, according to the default conditions by Gaussian. To obtain the solvation free energies, CPCM, IPCM, and PCM models were tested (Liptak et al., 2002). The pKa values were determined using Equations (4) and (5) and were corrected according to Pliego using Equation (6) (Pliego, 2003).

(4) $$\begin{array}{l} \Delta {\text{G}}_{sol}\;=\;\Delta {\text{G}}_{g\;}+\;\Delta {\text{G}}_{solv}\;\left({\text{A}}^{-}\right)\\ \;+\Delta \;{\text{G}}_{solv}\;\left(H_{3}O^{+}\right)\;-\Delta \;{\text{G}}_{solv}\;\left(HA\right)\\ \;-{\text{G}}_{solv}\left(H_{2}O\right) \end{array}$$

(5) $$pK_{a\;}\;=\;\frac{\Delta G_{sol}\;}{1.364}\;-\;log\left[H_{2}O\right]$$

(6) pKa(corrected) = pKa(calculated) − 4.54

## Results and discussion

### Spectrophotometric pKa determination

From the DHB spectra obtained at different pH (Figure S1 in Supplementary Information), we searched for an isosbestic point where the pKa was determined. For most of the DHBs, it was not possible to observe the isosbestic points directly from the data obtained by the spectrophotometer (C.S). Derivative spectrophotometry (D.S) must have been applied to the data to find the isosbestic points. Even different methods were used in combination with C.S or D.S to determine the pKa values. The method used to determine each pKa reported in this work is detailed in Table 2, and examples of the isosbestic points found for some DHBs are in Figure S2 in Supplementary Information.

**Table 2:** Method and wavelength used to determine each pKa value for all the DHBs studied.

	**Compound**	**pKa**	**λ (nm)**	**Methods**
1	4-Tert-butylcatechol	pKa_1_	297	C.S (Baseline to peak)
		pKa_2_	280	C.S (Baseline to peak)
2	4-Methylcatechol	pKa_1_	276, 293	C.S (Isosbestic point)
		pKa_2_	272	C.S (Baseline to peak)
3	4-Ethylcatechol	pKa_1_	286, 294	D.S (1° Der) (Isosbestic point)
		pKa_2_	275	C.S (Baseline to peak)
4	Hydrocaffeic acid	pKa_1_	206, 230	C.S (Isosbestic point)
		pKa_2_	288, 302	D.S (1° Der) (Isosbestic point)
		pKa_3_	194	C.S. (Seok)
5	3,4-Dihydroxyphenylacetic acid	pKa_1_	220, 229	D.S (1° Der) (Isosbestic point)
		pKa_2_	216, 230	D.S (1° Der) (Isosbestic point)
		pKa_3_	210, 223	D.S (1° Der) (Isosbestic point)
6	Catechol	pKa_1_	275, 293	C.S (Baseline to peak)
		pKa_2_	228, 240	D.S (1° Der) (Isosbestic point)
7	Norepinephrine	pKa_1_	288	C.S (Seok)
		pKa_2_	243	C.S (Seok)
		pKa_3_	311	C.S (Seok)
8	1,2,4-Benzenetriol	pKa_1_	284, 308	D.S (1° Der) (Isosbestic point)
		pKa_2_	335, 430	C.S (Isosbestic point)
		pKa_3_	405, 450	C.S (Isosbestic point)
9	Caffeic acid	pKa_1_	325, 360	D.S (1° Der) (Isosbestic point)
		pKa_2_	245, 270	D.S (1° Der) (Isosbestic point)
		pKa_3_	262, 280	D.S (1° Der) (Isosbestic point)
10	Dopamine	pKa_1_	287	C.S (Baseline to peak)
		pKa_2_	260	C.S (Baseline to peak)
11	4-Chlorocatechol	pKa_1_	233, 243	D.S (2° Der) (Isosbestic point)
		pKa_2_	261, 292	D.S (1° Der) Zero Crossing
12	Epinephrine	pKa_1_	287	C.S (Seok)
		pKa_2_	287	C.S (Seok)
13	3,4-Dihydroxybenzaldehyde	pKa_1_	206, 214	D.S (1° Der) (Isosbestic point)
		pKa_2_	246, 256	C.S (Isosbestic point)
14	3,4-Dihydroxybenzoic acid	pKa_1_	300, 310	D.S (1° Der) (Isosbestic point)
		pKa_2_	205, 216	D.S (1° Der) (Isosbestic point)
		pKa_3_	265, 277	C.S (Isosbestic point)
15	3,4-Dihydroxybenzylamine	pKa_1_	235, 250	C.S (Isosbestic point)
		pKa_2_	207, 216	D.S (1° Der) (Isosbestic point)
		pKa_3_	214, 220	D.S (1° Der) (Isosbestic point)
16	3,4-Dihydroxybenzonitrile	pKa_1_	252, 266	C.S (Isosbestic point)
		pKa_2_	250, 260	C.S (Isosbestic point)
17	4-Nitrocatechol	pKa_1_	258, 265	D.S (1° Der) (Isosbestic point)
		pKa_2_	294, 340	C.S (Isosbestic point)
18	Adrenalone	pKa_1_	281, 306	C.S (Isosbestic point)
		pKa_2_	253, 280	C.S (Isosbestic point)
		pKa_3_	264, 290	C.S (Isosbestic point)
19	3,4-Dihydroxyphenylalanine	pKa_1_	208, 232	C.S (Isosbestic point)
		pKa_2_	244	C.S (Seok)
		pKa_3_	291	C.S (Seok)
20	Catechin	pKa_1_	286	C.S (Baseline to peak)
		pKa_2_	286	C.S (Baseline to peak)
		pKa_3_	286	C.S (Baseline to peak)
21	Quercetin	pKa_1_	201, 210	D.S (1° Der) (Isosbestic point)
		pKa_2_	330, 360	C.S (Isosbestic point)
		pKa_3_	272, 290	C.S (Isosbestic point)
22	Chlorogenic acid	pKa_1_	216, 226	D.S (1° Der) (Isosbestic point)
		pKa_2_	335, 357	D.S (2° Der) (Isosbestic point)
23	Isoprenaline	pKa_1_	287	C.S (Baseline to peak)
		pKa_2_	221, 241	D.S (1° Der) (Isosbestic point)
24	Nordihydroguaiaretic acid	pKa_1_	288	C.S (Seok)

^*^C.S, classic spectrophotometry; D.S, derivative spectrophotometry.

The pKa values for the first and second dissociations for all the DHBs studied are detailed in Table 3. The results were obtained by spectrophotometric methods and these are in agreement with the results obtained by other researchers despite using different methodologies (Taulier et al., 1987; Erskine and Bobbitt, 1989; El-Sayed and El-Salem, 2005; Fernandes Previdello et al., 2006). For the nordihydroguaiaretic acid, only the first pKa value was determined because at high pH values a precipitate was formed.

**Table 3:** pKa values obtained by spectrophotometric method.

	**Compound**	**Experimental**		
		**pKa_1_**	**pKa_2_**	**pKa_3_**
1	4-*Tert*-butylcatechol	8.83 ± 0.02	13.93 ± 0.05	–
2	4-Methylcatechol	9.36 ± 0.01	13.49 ± 0.06	–
3	4-Ethylcatechol	8.32 ± 0.03	13.53 ± 0.05	–
4	3,4-Dihydroxydihydrocinnamic acid	4.86 ± 0.07	6.97 ± 0.02	11.75 ± 0.01
5	3,4-Dihydroxyphenylacetic acid	5.18 ± 0.04	10.04 ± 0.07	12.01 ± 0.04
6	Catechol	8.83 ± 0.03	13.07 ± 0.05	–
7	Norepinephrine	8.63 ± 0.06	10.60 ± 0.08	12.94 ± 0.03
8	1,2,4-Benzenetriol	9.24 ± 0.05	11.04 ± 0.04	12.91 ± 0.03
9	Caffeic acid	3.95 ± 0.03	8.47 ± 0.04	12.56 ± 0.05
10	Dopamine	8.91 ± 0.02	10.62 ± 0.06	12.67 ± 0.04
11	4-Chlorocatechol	7.90 ± 0.02	10.35 ± 0.03	–
12	Epinephrine	8.63 ± 0.04	10.90 ± 0.05	12.61 ± 0.07
13	3,4-Dihydroxybenzaldehyde	8.76 ± 0.05	11.77 ± 0.07	—
14	3,4-Dihydroxybenzoic acid	3.17 ± 0.09	9.10 ± 0.08	11.77 ± 0.05
15	3,4-Dihydroxybenzylamine	5.67 ± 0.08	8.85 ± 0.06	12.50 ± 0.04
16	3,4-Dihydroxybenzonitrile	6.47 ± 0.02	11.71 ± 0.05	–
17	4-Nitrocatechol	5.93 ± 0.05	11.05 ± 0.04	–
18	Adrenalone	4.34 ± 0.04	6.43 ± 0.06	11.85 ± 0.02
19	Dopa	2.06 ± 0.06	8.55 ± 0.06	9.93 ± 0.03
20	Catechin	8.67 ± 0.07	9.37 ± 0.05	11.60 ± 0.02
21	Quercetin	6.76 ± 0.04	9.10 ± 0.03	11.26 ± 0.05
22	Chlorogenic acid	3.36 ± 0.05	8.10 ± 0.04	11.51 ± 0.04
23	Isoprenaline	8.24 ± 0.07	9.99 ± 0.06	–
24	Nordihydroguaiaretic acid	9.38 ± 0.04	–	–

### Computational pKa determination

The computational calculations were obtained using the 6-311+G(d,p) basis set. In the present work, the basis set used for catechol (Altarawneh et al., 2010) was extrapolated for the structure optimization of different DHBs substituted in 4-position. On the other hand, to obtain computational pKa values (comparable with the experimental data), different solvation models in water were tested and are showed in Table 4. From the comparison of these values, the relative error was determined. The relative error was higher when the pKa was calculated using IPCM model (2.83% error in catechol and 13.5% error in 4-nitrocatechol determination). The smallest relative error was observed with the CMPC solvation model and this was not higher than 3%.

**Table 4:** Comparison of pKa values obtained experimentally and computationally with different solvation models.

**Compound**	**First pKa value calculated**			
	**Experimental**	**PCM model**	**IPCM model**	**CPCM model**
Catechol	8.83	8.77	8.58	8.82
4-Nitrocatechol	5.93	5.58	5.13	6.10
4-Ethylcatechol	8.29	8.44	8.89	8.20

**Figure 4:**
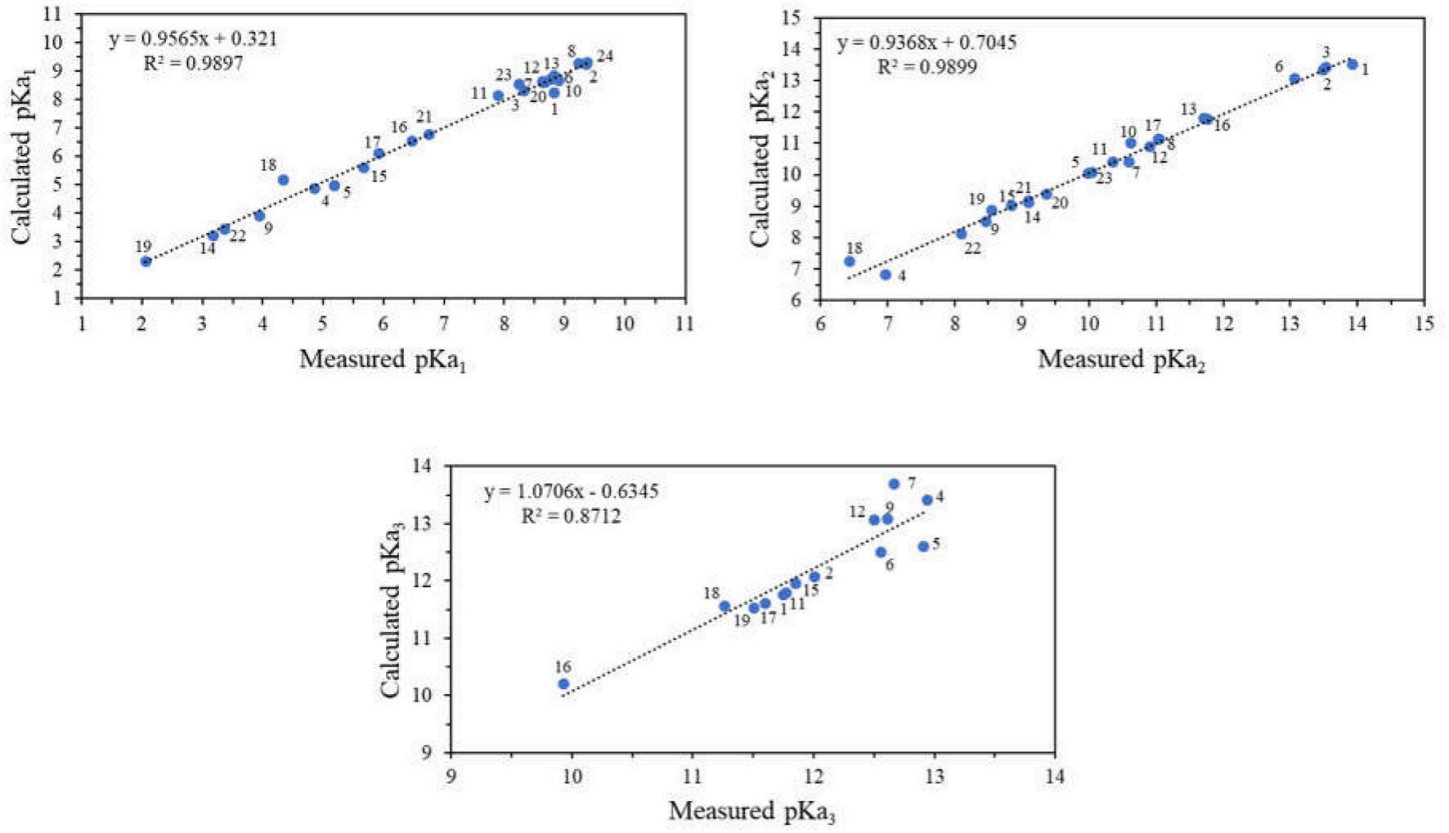
Correlation diagram of pKa values for different DHBs substituted on 4-position obtained experimentally and computationally.

For all the DHBs under the study, the pKa values in aqueous solution were calculated at DFT level with the CPCM solvation model, and this was in agreement with calculations in similar compounds (Liptak et al., 2002). Figure 4 shows that the calculated pKa values exhibited good accuracy with those pKa determined experimentally. The correlation (r^2^) for each pKa determined was higher than 0.87. Also, it was possible to calculate the pKa_2_ for nordihydroguaiaretic acid through computational calculation, which was not possible to determine experimentally due to limitations of the technique.

**Figure 5:**
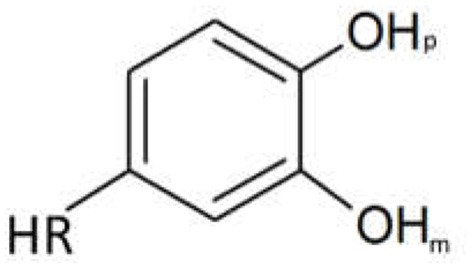
DHBs labels used in this paper.

The determination of each computational pKa allows the identification of the outgoing proton for each value, respectively. To simplify the analysis, Figure 5 shows that the pKa values determined were classified as part of one of the neighboring hydroxyls being the hydroxyl group in meta position (OH_m_) or para position (OH_p_) with respect to the substituent or as part of the substituent group (RH).

The pKa whose value was less than 5.0 corresponds to the deprotonation equilibrium of carboxylic acids (RH). These results agree with the greater stability expected for these unprotoned groups (Clayden et al., 2012). In this context, different acidity influence was obtained in the DHBs studied due to their different substituent on 4-position. Hence, some of them can be classified as a donor of electron density (EDG) or as an electron density withdrawing group (EWG).

The Hammett constants (σ) reported for these groups are shown in Table 5. The DHBs with EDG have σ < 0 and are over the catechol, and below the catechol are the DHBs with EWG, which have σ > 0.

**Table 5:** pKa values obtained for different DHBs determined by computational method and the Hammett constant (σ) of each substituent on 4 position.

	**Compound**	**Computational**			
		**pKa(OH_m_)**	**pKa(OH_p_)**	**pKa(RH)**	**σ_p_**
1	4-*Tert*-butylcatechol	8.24	13.51	–	−0.2
2	4-Methylcatechol	9.23	13.33	–	−0.17
3	4-Ethylcatechol	8.29	13.42	–	−0.15
4	3,4-Dihydroxydihydrocinnamic acid	6.83	11.76	4.88	−0.07
5	3,4-Dihydroxyphenylacetic acid	10.09	12.07	4.98	
6	Catechol	8.82	13.07	–	0
7	Norepinephrine	13.41	8.59	10.41	0.09
8	1,2,4-Benzenetriol	9.26	11.13	12.60	−0.37
9	Caffeic acid	12.50	8.50	3.92	0.09
10	Dopamine	13.7	8.66	11.01	0.17
11	4-Chlorocatechol	8.13	10.41	–	0.23
12	Epinephrine	13.08	8.65	10.89	0.3
13	3,4-Dihydroxybenzaldehyde	11.78	8.71	–	0.42
14	3,4-Dihydroxybenzoic acid	11.79	9.11	3.22	0.45
15	3,4-Dihydroxybenzylamine	13.07	5.62	9.01	0.53
16	3,4-Dihydroxybenzonitrile	11.79	6.54	–	0.66
17	4-Nitrocatechol	11.15	6.10	–	0.78
18	Adrenalone	11.96	5.16	7.24	
19	Dopa	13.6	8.86	2.31/10.2^*^	
20	Catechin	8.61	9.38	11.61	
21	Quercetin	6.78	11.56	9.17	
22	Chlorogenic acid	11.53	8.11	3.43	
23	Isoprenaline	8.52	10.06		
24	Nordihydroguaiaretic acid	9.30	14.31		

* *This compound was deprotonated in two groups in the substituent*.

**Figure 6:**
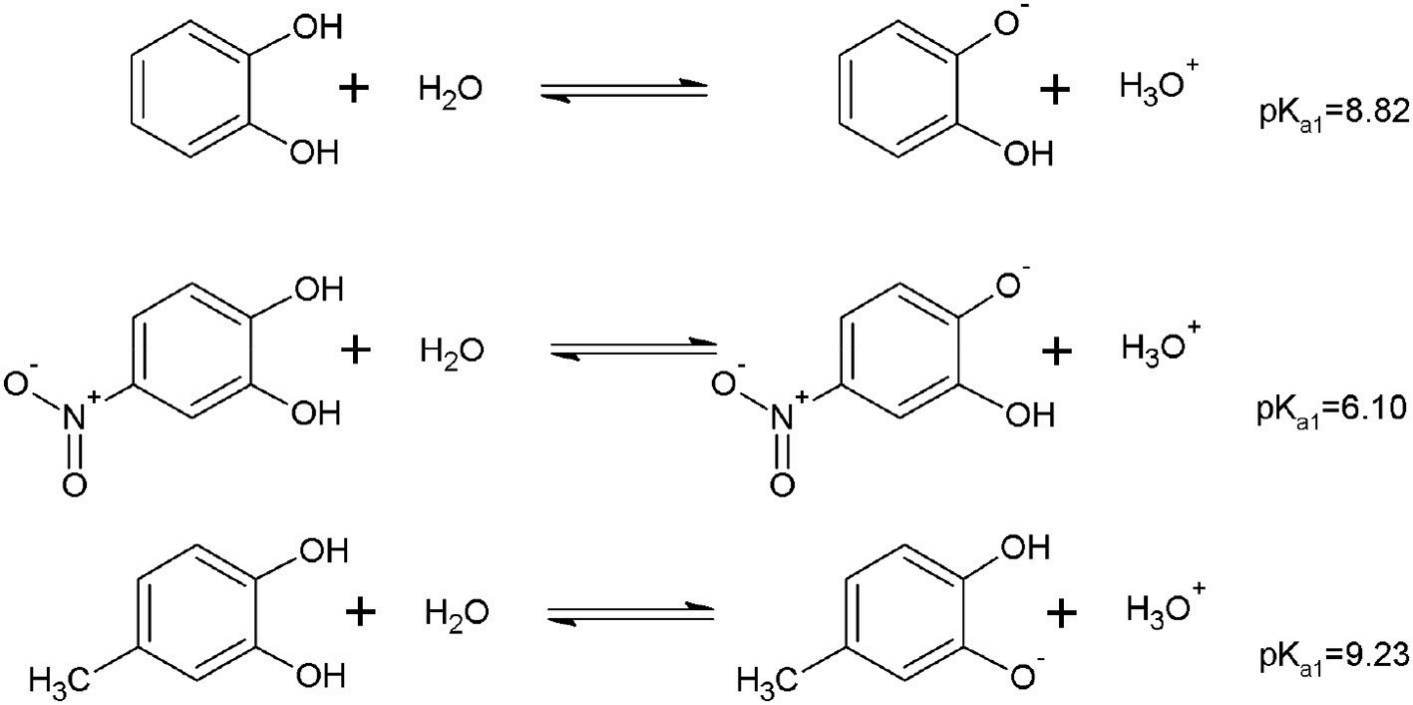
Example of the equilibrium associated to the pKa for the first hydroxyl deprotonation of DHBs substituted with EDG and EWG.

Figure 6 shows that according to the computational calculations, the more acidic proton was the OH_m_ for DHBs with EDG. Otherwise, in the DHBs with EWG, the more acidic proton was in the OH_p_. The substituent can contribute to the charge stabilization of the anion that is formed by losing a proton. For DHBs with EWG, the anion formed is stabilized by resonance. In consequence, the OH_p_ shows lower pKa values than the DHBs with EDG.

**Figure 7:**
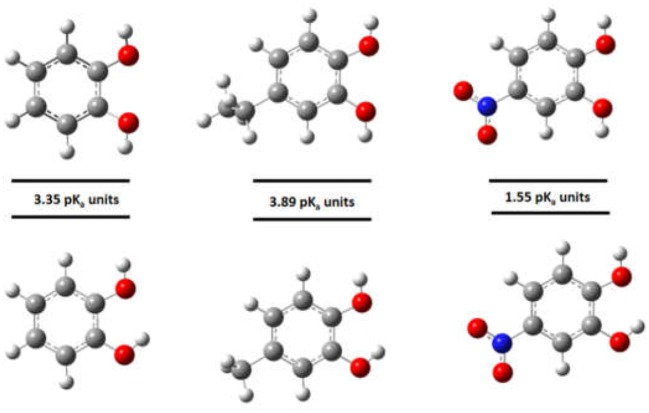
Effect of the intramolecular hydrogen bond in the pKa calculations.

Moreover, it is well-known that in the optimization of a DHB structure, an intramolecular hydrogen bond must be considered between the neighboring hydroxyl groups to achieve good results (Zhu et al., 2010). As an example, Figure 7 shows that when the hydrogen bond was not considered in the computational pKa determination, the values were significantly different than the experimentally determined pKa. The difference between the calculated pKa with or without hydrogen bonding is higher for DHBs substituted by EDG than DHBs substituted by EWG, and this is explained by the charge stabilization of the molecule with substituents with higher electronegativity.

In general, for the DHBs substituted by a EWG, the first deprotonated hydroxyl group is OH_p_. On the other hand, if the DHBs are substituted by EDG (and for catechol), then the first deprotonated hydroxyl group is OH_m_.

It has been noted that 4-chlorocatechol and 1,2,4-benzenotriol do not conform to this trend. This behavior could be explained because both chlorine and hydroxyl groups are electron-withdrawing, because of their electronegativity, but they are electron-donating by resonance. This effect is higher for hydroxyl groups because the overlapping between 2p and 2p orbitals is higher than the overlapping between 2p and 3p orbitals of chlorine (Clayden et al., 2012). In this way, the hydroxyl and chlorine are considered weak deactivating substituents. Therefore, for the 4-chlorocatechol and 1,2,4-benzenetriol, the first deprotonation of hydroxyl groups was OH_m_.

## Conclusion

In this work, an experimental validated computational method was implemented in aqueous solution for pKa determination of different DHBs, which are high value compounds and have had a great attention for its use as versatile platforms for the design of different materials. Also, it is well-known for its metal ligands properties with pro-oxidant and antioxidant performance in biological systems. In this framework, the knowledge of pKa is useful to explain the differences in the reactivity of DHBs compounds in their metallic complexing and subsequent activity in AOPs and biological systems.

Therefore, the pKa were calculated at DFT level with CPCM solvation model due to its relative error which was lower than 3% compared to the obtained experimental data. Furthermore, it was possible to predict the pKa value for each outgoing proton in the DHB structure, which was explained based on donor of electron density (EDG) or electron density withdrawing group (EWG) of each substituent in the DHB structure.

## Author contributions

RR performed the computational calculation of pKa values. PS performed the experimental determination of pKa values. CS supervised the experiments and the data treatment to determine the pKa values experimentally. VM and DC wrote the manuscript.

### Conflict of interest statement

The authors declare that the research was conducted in the absence of any commercial or financial relationships that could be construed as a potential conflict of interest.
