# Supplementary material for: An Experimental Validated Computational Method for pKa Determination of Substituted 1,2-Dihydroxybenzenes
Source: Front Chem. 2018 Jul 13;6:208. doi: 10.3389/fchem.2018.00208 (PMC6053874; doi:10.3389/fchem.2018.00208)
Supplement: Supplementary file 1 [file Image_1.PDF]

Supplementary Material

An experimental validated computational method for pKa determination of substituted 1, 2-dihydroxybenzenes.

Romina Romero[a], Pablo Salgado[b], César Soto[d], David Contreras[c,d], Victoria Melin[c*]

Correspondence: Dr. Victoria Melin Coloma: victoriamelin@udec.cl

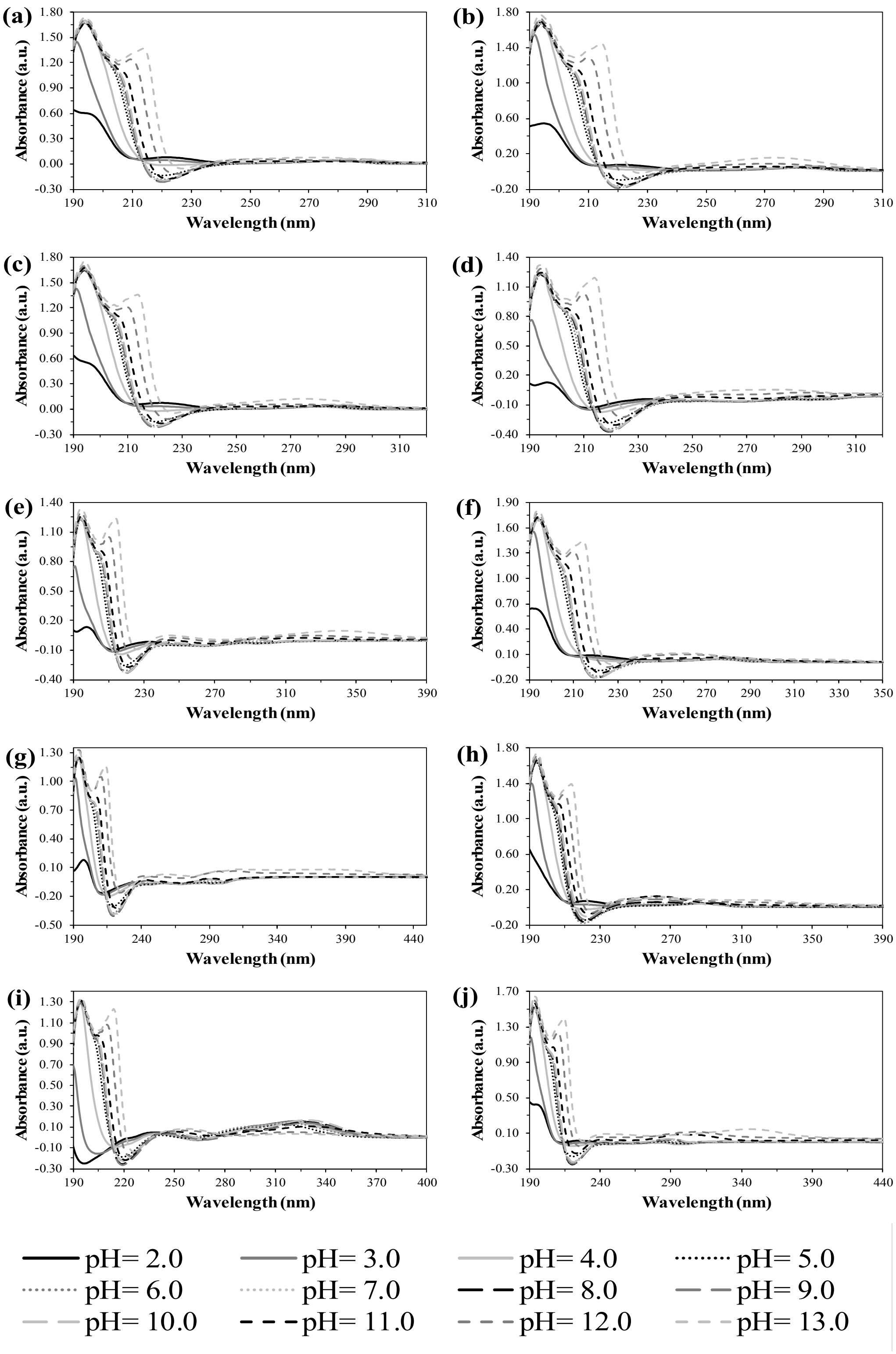

Figure S1. DHBs spectrum at different pH. a) 4-terbutylcatechol, b) 4-methylcatechol, c) 4-ethylcatechol, d) 3,4-dihydroxyhydrocinnamic acid, e) 3,4-dihydroxyphenylacetic acid, f) catechol, g) norepinephrine, h) 1,2,4-benzenetriol, i) caffeic acid.

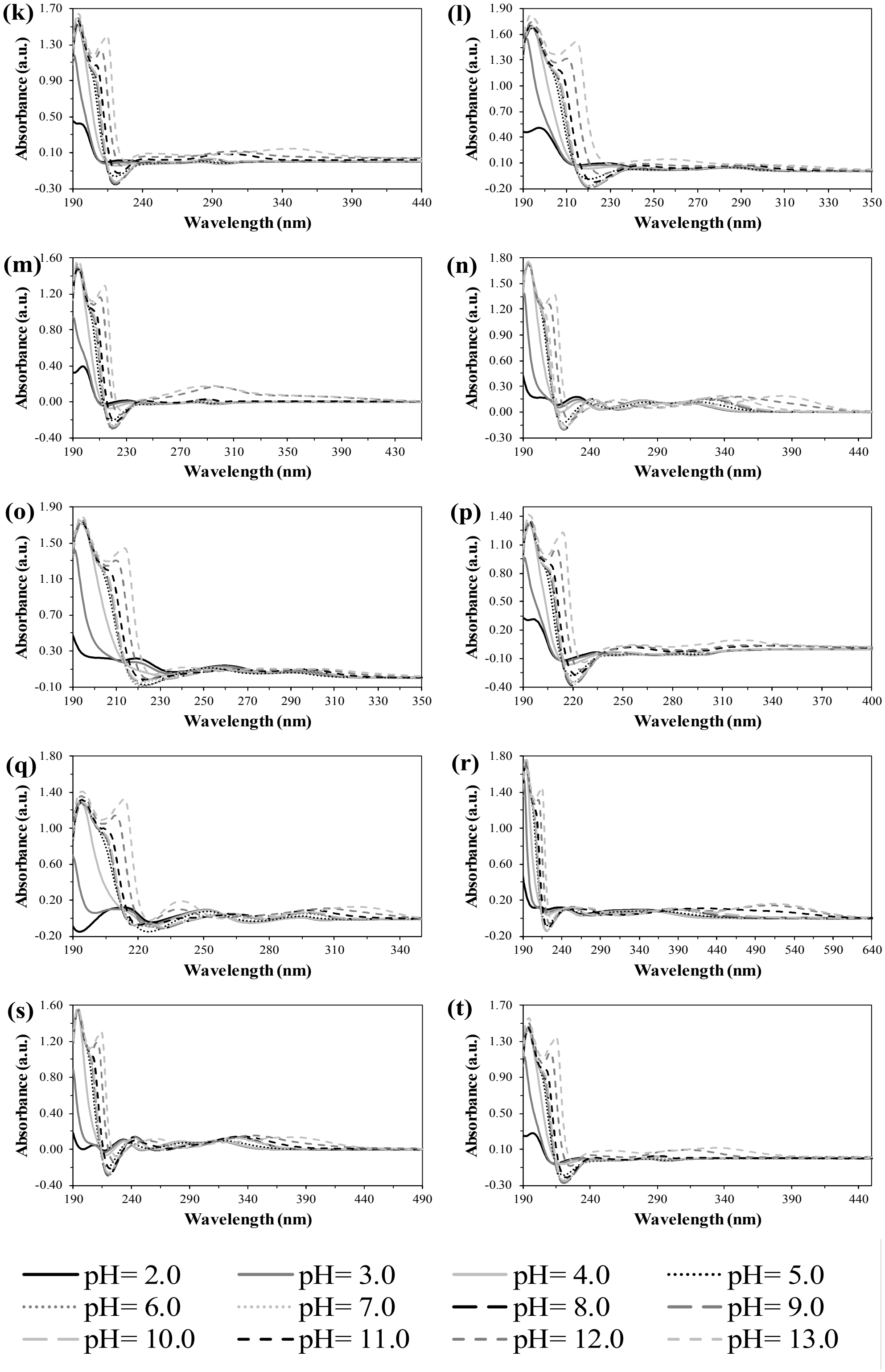

Figure S1. DHBs spectrum at different pH. k) dopamine, l) 4-chlorocatechol, m) epinephrine, n) 3,4-dihydroxybenzaldehyde, o) 3,4-dihydroxybenzoic acid, p) 3,4-dihydroxybencylamine, q) 3,4-dihydroxybenzonitrile, r) 4-nitrocatechol, s) adrenalone, t) DOPA.

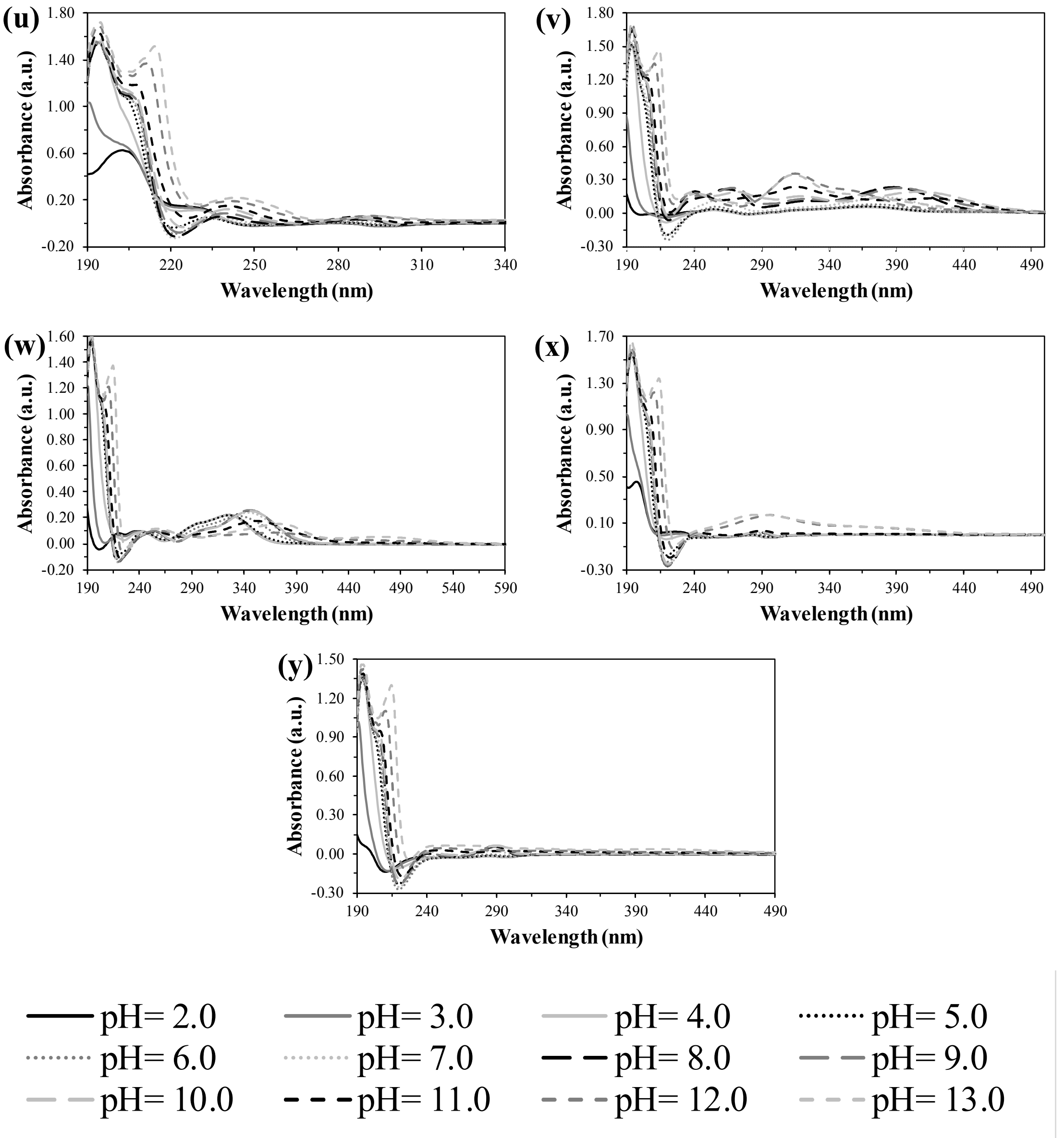

Figure S1. DHBs spectrum at different pH. u) catechin, v) quercetin, w) chlorogenic acid, x) isoprenaline, y) nordihydroguaiaretic acid.

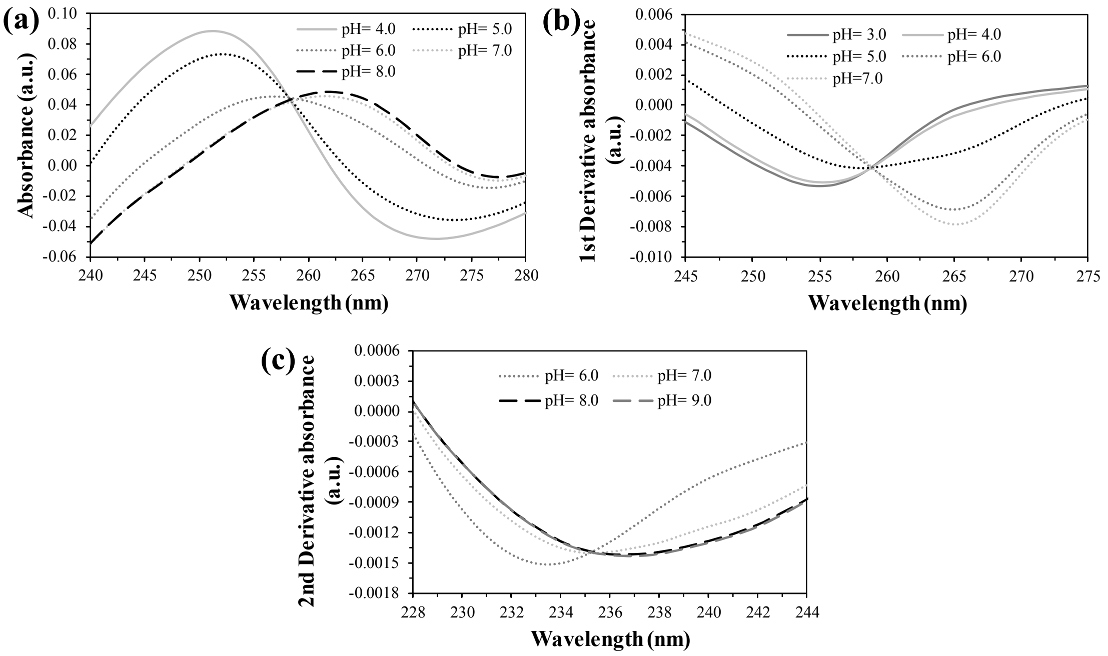

Figure S2. Examples of isosbestic point obtained by applied different methods at the experimental data a) pKa_1 of 3,4-dihydroxybenzonitrile obtained by classic spectrophotometric b) pKa_1 of 4-nitrocatechol obtained by derivative spectrophotometry (1° derivative) and c) pKa_1 of 4-chlorocatechol obtained by derivative spectrophotometry (2° derivative).
